# Access to Hearing Healthcare and Barriers Among United States Veterans: A Survey‐Based Study

**DOI:** 10.1002/oto2.70055

**Published:** 2025-01-09

**Authors:** Tyler J. Gallagher, Kaitlin Hori, Janet S. Choi

**Affiliations:** ^1^ Keck School of Medicine of the University of Southern California Los Angeles California USA; ^2^ Caruso Department of Otolaryngology–Head and Neck Surgery Keck School of Medicine of the University of Southern California Los Angeles California USA

**Keywords:** access, audiology, hearing, veterans

## Abstract

**Objective:**

We investigated use of hearing care among US veterans and explore motivations, barriers, and adherence to hearing healthcare.

**Study Design:**

Cross‐sectional online survey.

**Setting:**

US Veterans.

**Methods:**

This cross‐sectional, online survey‐based study included 287 respondents who identified themselves as veterans on ResearchMatch (NIH‐sponsored national registry of research volunteers). Questions regarding hearing loss, tinnitus, use of hearing care, and facilitators/barriers to hearing care were asked. Descriptive statistics were utilized for data analysis.

**Results:**

In this cohort, the rate of self‐reported hearing loss and tinnitus were 61.0% and 74.2%, respectively. Among these, 66.9% of those with hearing loss and 36.2% of those with tinnitus reported seeking hearing care. Most important motivators of seeking hearing care (average on 1‐5 Likert scale) included personal importance of hearing (4.0; SD = 1.1), difficulty hearing others (3.6; SD = 1.1), and degree of hearing loss (3.3; SD = 1.1). Most common barriers included uncertainty regarding who to reach out to for hearing care (42.9%), inability to get time off work (34.3%), and nervousness about seeing a provider (28.6%). Common reasons to decline hearing aids reporting that hearing was not bad enough for hearing aids (72.2%) and included inability to afford hearing aid (55.6%).

**Conclusion:**

In this cohort, many US veterans sought care for hearing loss, though barriers still exist, including uncertainty regarding how to access appropriate care, being too far from a provider, and challenges with cost. Future studies should investigate methods to alleviate these internal and external barriers to hearing care and emphasize the importance of motivators for seeking care.

There are approximately 17 million veterans of the US Armed Forces, comprising 5% of the US population; among veterans, hearing loss and tinnitus represent the two most common disabilities compensated by the Veterans Benefits Administration.^1^, ^2^ More than 1.3 million veterans receive disability compensation for hearing loss and more than 2.3 million are compensated for tinnitus annually.^2^ To receive evaluation and treatment of hearing loss, US veterans are required to register and verify service status at their local VA, then schedule an appointment for audiologic evaluation and, if necessary, hearing aid fitting.^3^ In line with national standards, there is no automatic hearing screening conducted for veterans despite high prevalence.^4^ Furthermore, award of free hearing aids requires a disability rating—a result of a separate process to demonstrate that hearing loss is substantial and due to service‐related requirements.^5^

Despite their high prevalence, hearing loss, and tinnitus are not among the top fifteen diseases seen in Veteran's Affairs (VA) health care utilization of veterans, emphasizing their low usage of hearing healthcare.^6^ Veterans are particularly susceptible to hearing health issues due to occupational exposure to loud noises and related hazards with higher prevalence among those with more years of military service, greater noise exposure, and exposures to blasts.^7^, ^8^, ^9^ Furthermore, rates of tinnitus among active duty service members have more than tripled from 2001 to 2015.^10^ These numbers will quickly translate into rising rates of hearing loss and tinnitus among veterans, which is particularly concerning due to demonstrated associations between hearing loss and tinnitus with mental health disorders, poorer cognitive function, and greater healthcare utilization.^8^, ^9^, ^11^, ^12^

Previous studies have examined access and utilization of hearing healthcare among the civilian US adult population. A systematic review has shown that facilitators to establish hearing care include degree of hearing loss, self‐efficacy towards hearing care (especially hearing aids), family support, and self‐recognition of hearing loss.^13^ Barriers to care include financial limitations, stigma of hearing devices, inconvenience, competing chronic health problems, and unrealistic expectations.^13^, ^14^ Finally, compliance to hearing healthcare is affected by self‐efficacy, education level, and engagement in rehabilitation.^14^ Compared to investigations of barriers to hearing care among the civilian population, there has been very sparse investigation of such barriers among veterans.

Prior studies have demonstrated barriers to hearing healthcare within the veteran population. When compared to the civilian population, veterans receive cochlear implants (CI) at an older age, have longer duration of hearing loss, and higher rates of pre‐operative hearing aid use.^15^ Many veterans also endure geographical barriers to receive medical care at a VA facility, as over half of veterans were estimated to live over 80 miles away from a covered facility.^16^ Furthermore, while tinnitus care also exists in the VA system, clinical protocols for its management are highly variable across VA systems.^17^ Due to the aforementioned barriers, many Veterans choose to utilize private insurance coverage for care needs, thereby losing out on VA benefits.^18^

In this survey‐based study, we aim to investigate various factors related to hearing healthcare utilization among veterans using a convenience sample of US veterans recruited through ResearchMatch. Here, we assess current utilization of hearing healthcare services, any delays in accessing such care, and factors that influence veterans’ decisions to pursue hearing care, including motivators, barriers, and compliance with hearing care recommendations.

## Methods

This is an online‐based cross‐sectional survey study. The study was reviewed by the University of Southern California Institutional Review Board (UP‐23‐01232).

### Study Cohort

Respondents were recruited via ResearchMatch—a national registry for research volunteer recruitment created and maintained by the National Institute of Health (NIH). Individuals were eligible for participation if they were an adult (≥18 years) reporting being a veteran. ResearchMatch is a database for both healthy and symptomatic volunteers consenting to be contacted by researchers about health studies. ResearchMatch was created by several academic institutions with the support of the NIH as part of the Clinical Translational Science Award program.^19^ ResearchMatch has been utilized to recruit individuals participants for survey‐based studies and randomized clinical trials alike.^20^, ^21^, ^22^ Response rate after recruitment was 3.2% (287/9079). While ResearchMatch has volunteers from all 50 states, the sample is not nationally representative.

### Survey Development and Distribution

An anonymous, survey of 5 to 15 minutes in length was designed using REDCap electronic data capture tools.^23^ Survey questions started with a brief demographic questionnaire, followed by questions regarding hearing loss and tinnitus symptoms via two validated questionnaires—the Hearing Handicap Inventory for Adults–Short Form (HHIA‐S)^24^ and tinnitus‐related questions from the Tinnitus Handicap Index (THI).^25^ As there were no validated questionnaires available to measure access to hearing care and their facilitators and barriers, our team of researchers developed a list of questions based on clinical experiences and literature review. These questions were designed to explore three outlined aspects of access—motivation, barriers, and adherence to care recommendations—that have previously been described.^13^ After iterative process of revision, the final survey had a minimum of 31 questions and a maximum of 94 questions (Supplement A, available online). Survey distribution occurred on March 18, 2024, and responses were collected until April 7, 2024. Upon completion, participants were eligible to enter a raffle for a $50 gift card.

### Demographic Characteristics

We collected information on the following demographic variables: age, gender, marital status, race/ethnicity, highest level of education, annual household income, and primary healthcare insurance type. All participants subsequently completed the HHIA‐S and THI, followed by individual questions querying self‐report of hearing loss, tinnitus, experience with hearing loss evaluation, and finally whether individuals sought care for hearing loss or tinnitus.

### Access to Hearing Care Questionnaires

Respondents received questions related to three domains of hearing healthcare access—motivation, barriers, and adherence to care recommendations. Regarding motivation, individuals were asked Likert‐scale questions to rate how important various factors were in their decision to seek care for hearing loss and/or tinnitus if they reported having trouble with hearing loss and/or tinnitus. Regarding barriers, individuals were asked Likert‐scale questions to rate how important a variety of barriers were in getting hearing care access if they sought hearing health care. Finally, regarding adherence to care recommendations, individuals were asked Likert‐scale questions to rate how important various factors were in their decision to decline use of a hearing aid or cochlear implant, when applicable. Finally, all participants were asked about their general familiarity with a variety of hearing rehabilitation devices.

### Statistical Analysis

Analytic cohort included respondents who completed the entire survey. The primary outcome was the rate of respondents who sought any hearing care among those reporting hearing loss and/or tinnitus. Secondary outcomes included motivation for seeking hearing healthcare, barriers to accessing hearing healthcare, adherence with recommended hearing interventions, and familiarity with a variety of hearing assist devices. Outcomes were analyzed via descriptive statistics. An ANOVA was utilized to describe the differences in means with a post‐hoc Bonferroni test to determine differences between groups. All analyses were completed in STATA Version 18.0 Standard Edition (StataCorp LLC). Significance was set at *P* < .05, two‐tailed.

## Results

Study cohort (n = 287) characteristics were summarized in Table 1. Our cohort were on average 61.0 years old (SD = 15.6 years), primarily male (68.6%), and White/Caucasian (87.5%). Most respondents reported being college graduates (n = 107, 37.3%), were primarily insured by a VA Health Plan or TRICARE (40.8%), reported annual income of $75,000 to 99,999 (18.8%), and were married (61.0%).

**Table 1:** Demographic Characteristics (n = 287)

Characteristic	Frequency (%)
Age (years)	
Mean (SD)	61.0 (15.6)
Range	24‐92
Sex	
Female	88 (30.7)
Male	197 (68.6)
Other	2 (0.7)
Race	
White/Caucasian	251 (87.5)
Hispanic/Latino	10 (3.5)
Black/African American	16 (5.6)
Other	10 (3.5)
Education	
High School Degree	9 (3.1)
Some College	62 (21.6)
College Graduate	107 (37.3)
Graduate School Degree	87 (30.3)
Doctoral Degree	22 (7.7)
Insurance	
VA Health Plan or TRICARE	117 (40.8)
Medicare	93 (32.4)
Medicaid	11 (3.8)
Private/Other Employee Sponsored	63 (21.9)
No Insurance	3 (1.1)
Income ($)	
<25k	17 (5.9)
25‐49,999	33 (11.5)
50‐74,999	51 (17.8)
75‐99,999	54 (18.8)
100‐149,999	52 (18.1)
150‐199,999	39 (13.6)
200k or >	39 (13.6)
Other	12 (4.2)
Marital status	
Married	157 (61.0)
Single	43 (15.0)
Long‐term partnership	14 (4.9)
Separated/divorced	48 (16.7)
Other	7 (2.4)
HHIA–S	
Mean (SD)	13.7 (11.8)
Range	0‐40
THS	
Mean (SD)	2.1 (3.3)
Range	0‐16

**Figure 1:**
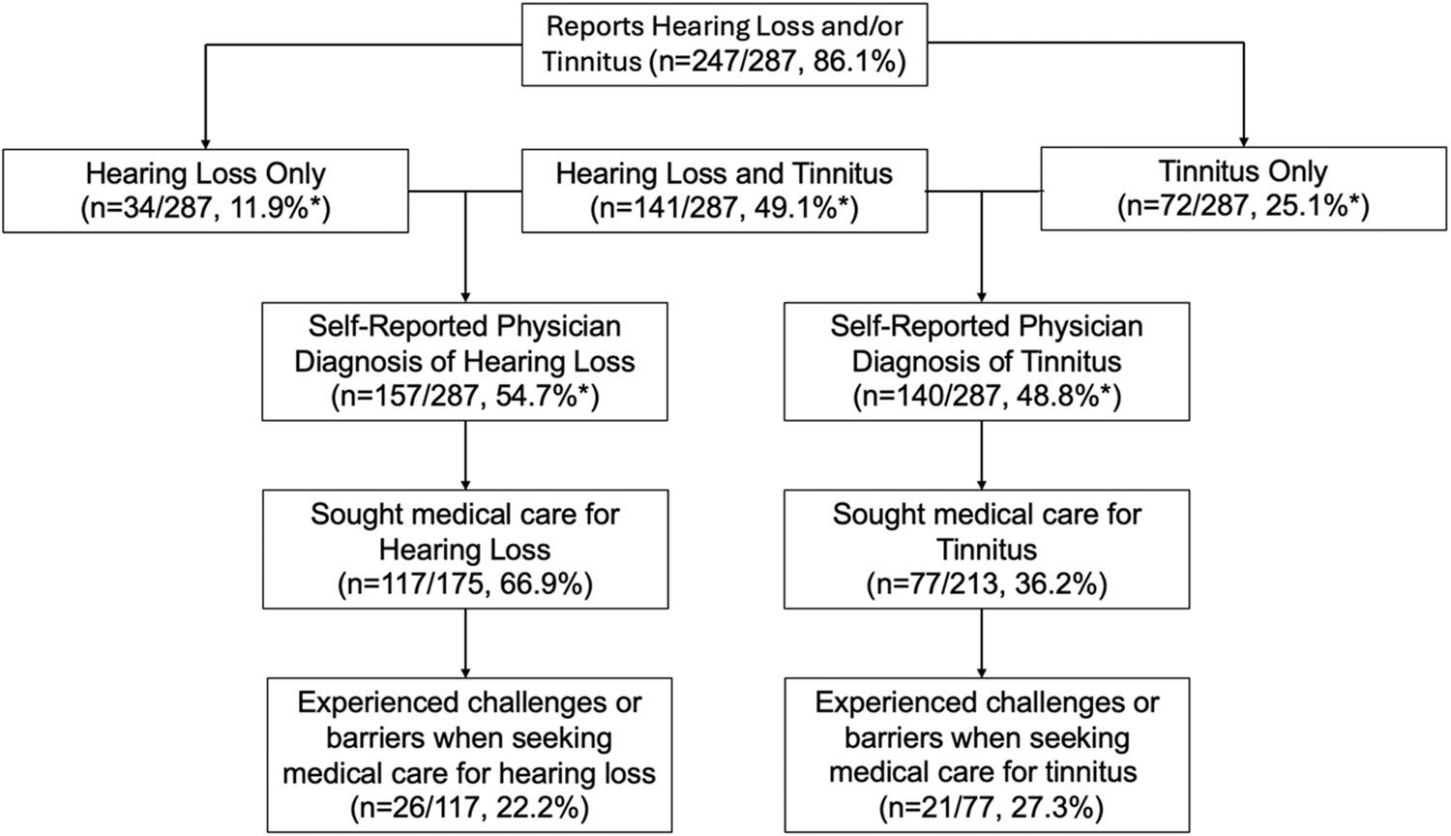
Tree of prevalence of HL/tinnitus, medical care seeking, barriers to care, and HA recommendation/utilization. *Percentages represent portion of all 287 respondents. Other percentages describe percent of the group reporting hearing loss and/or tinnitus.

In this cohort, the mean score of HHIA‐S (score range 0‐40) was 13.7 (SD = 11.8) and the mean scores of THI (score range 0‐16) was 2.1 (SD = 3.3). This indicates an average light‐to‐moderate hearing handicap and slight or no tinnitus handicap. Figure 1 describes prevalence of self‐report of issues with hearing loss and tinnitus, as well as rates of seeking care and experiencing barriers to care. Most of the cohort (86%) reported having hearing loss and/or tinnitus with a half of the cohort reporting both hearing loss and tinnitus (49.1%). Among those self‐reporting hearing loss, 66.9% sought medical care for hearing loss, and 22.2% of those individuals reported experiencing challenges or barriers when seeking medical care for hearing loss. Among those self‐reporting tinnitus, 36.2% reported seeking medical care for tinnitus and 27.3% of those individuals reported experiencing challenges or barriers to when seeking medical care for tinnitus.

Among individuals reporting ever having an audiogram (n = 267; 93.0%), the average time from most recent audiogram was 8.0 (SD = 9.3) years ago. Among those reporting hearing loss, the duration of hearing loss was, on average, 17.9 (SD = 13.1) years, and individuals sought care on average 9.7 (SD = 12.9) years after noticing hearing loss. Among those reporting tinnitus, the duration of tinnitus was, on average, 19.0 (SD = 14.2) years ago, and individuals sought care on average 7.4 (SD = 9.6) years after noticing tinnitus.

**Figure 2:**
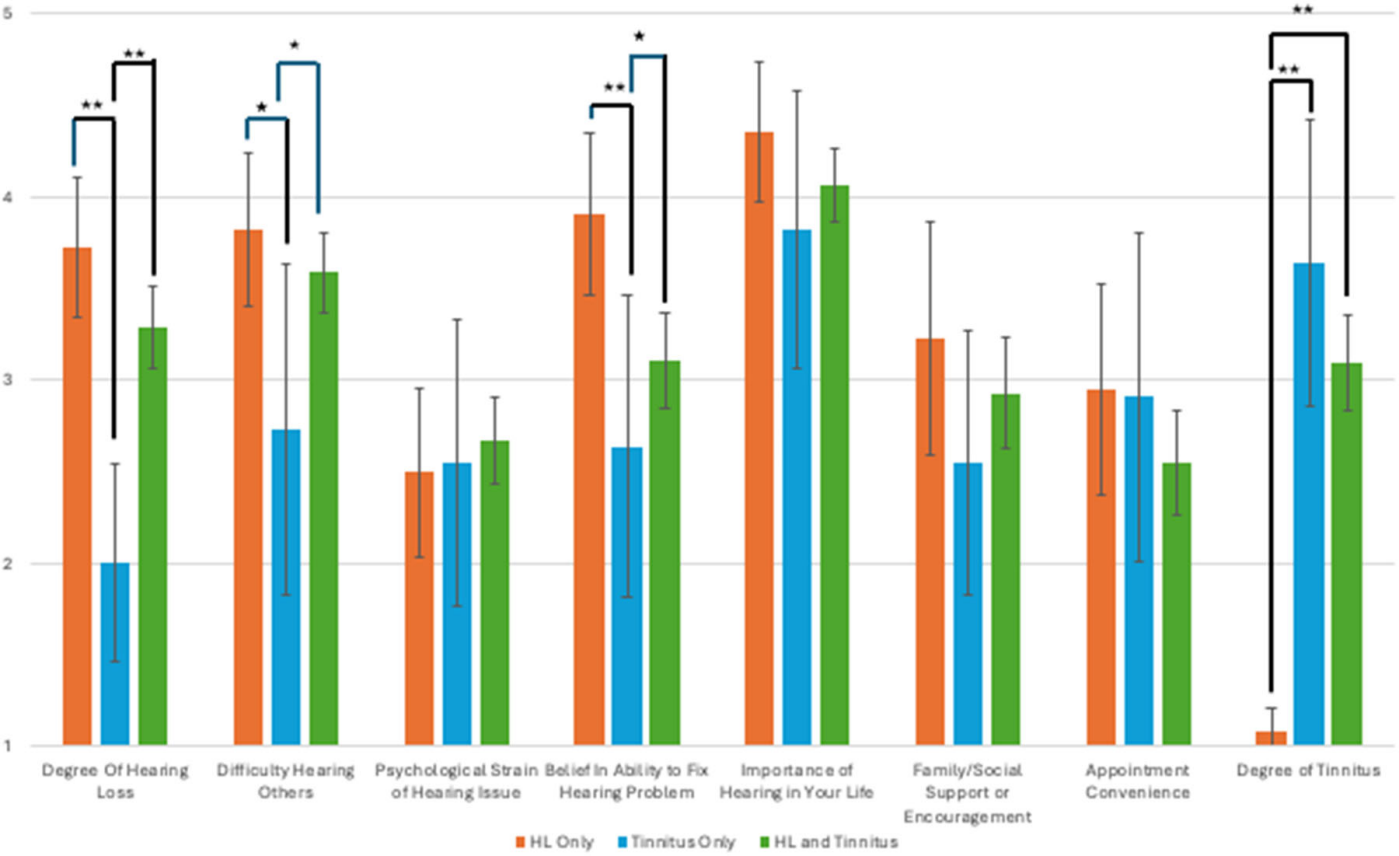
Contribution of various factors to motivation for seeking hearing and/or tinnitus care. *P < .05, **P < .01.

Figure 2 depicts respondent‐rated factors that contributed to their decisions in seeking hearing care. Among the respondents who reported hearing loss, the top three highest‐rated factors that contributed to their decision in seeking care for hearing loss were (1‐5 Likert scale): (1) importance of hearing in their life (4.4 [95% CI: 4.0‐4.8]), (2) belief in ability to fix hearing problem (3.9 [95% CI: 3.5‐4.4]), and (3) difficulty hearing others (3.8 [95% CI: 3.4‐4.3]). For the respondents who reported tinnitus, the top three highest‐rated factors for seeking care for tinnitus were: (1) importance of hearing in their life (3.8 [95% CI: 3.0‐4.7]), (2) degree of tinnitus (3.6 [95% CI: 2.8‐4.5]), and (3) appointment convenience (2.9 [95% CI: 1.9‐3.9]). Lastly, for those with both hearing loss and tinnitus, the most common reasons were importance of hearing in their life (4.1 [95% CI: 3.9‐4.3]), difficulty hearing others (3.6 [95% CI: 3.4‐3.8]), and appointment convenience (3.4 [95% CI: 3.2‐3.6]).

**Figure 3:**
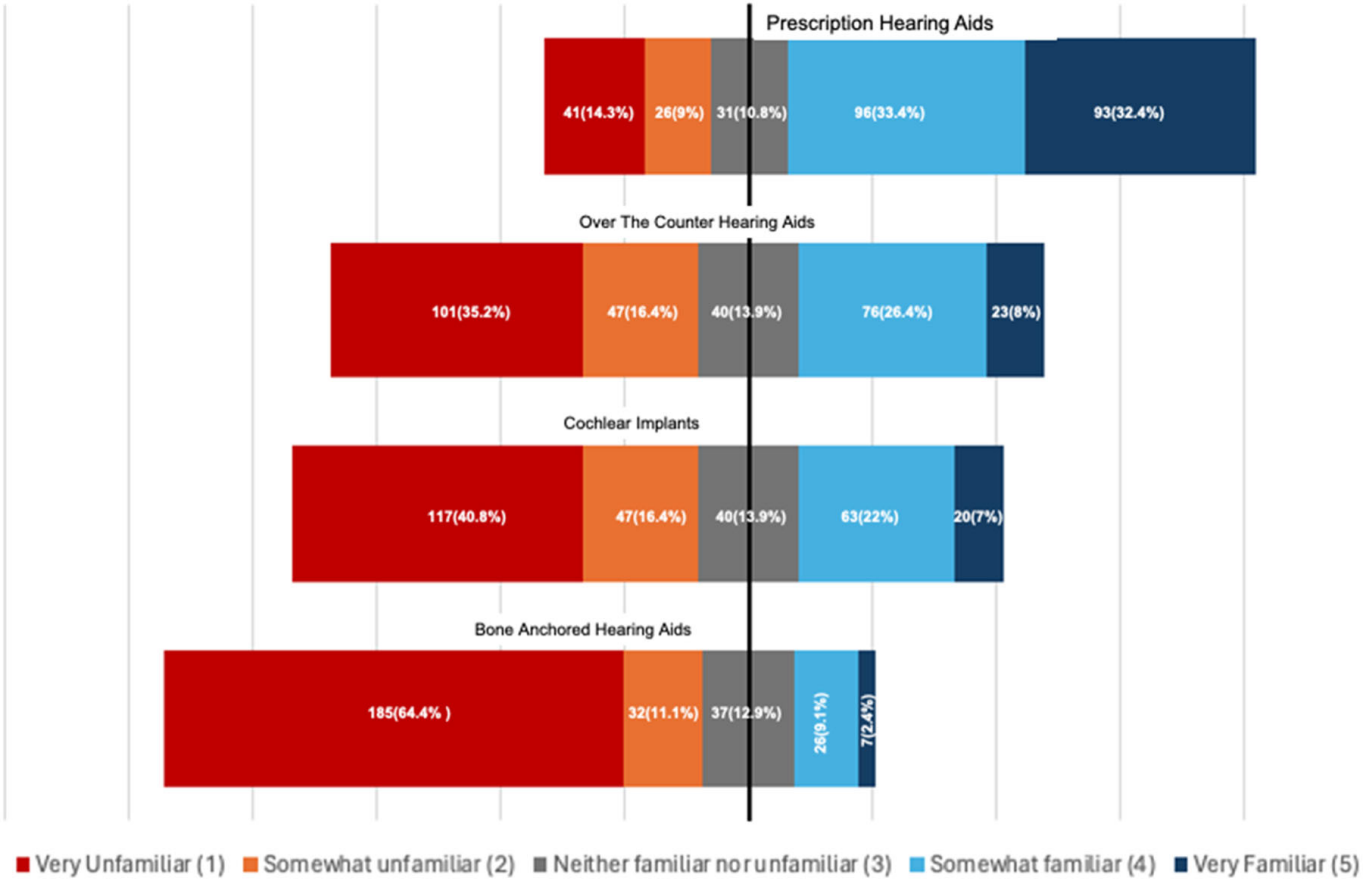
Familiarity with various hearing loss treatments*. *Familiarity is described by Likert scale response to the question “How familiar are you with the following treatments for hearing?”

To evaluate awareness of various hearing rehabilitative options that may influence decision to pursue hearing care among all Veterans, we evaluated familiarity with various hearing rehabilitation options utilizing a 5‐point Likert scale (Figure 3). The most common level of familiarity with prescription hearing aids was somewhat familiar (33.4%) closely followed by very familiar (32.4%). Many respondents were unfamiliar with hearing rehabilitation options other than conventional hearing aids including over‐the‐counter hearing aids (35.2%), cochlear implants (40.8%), and bone‐anchored hearing aids (64.4%).

**Figure 4:**
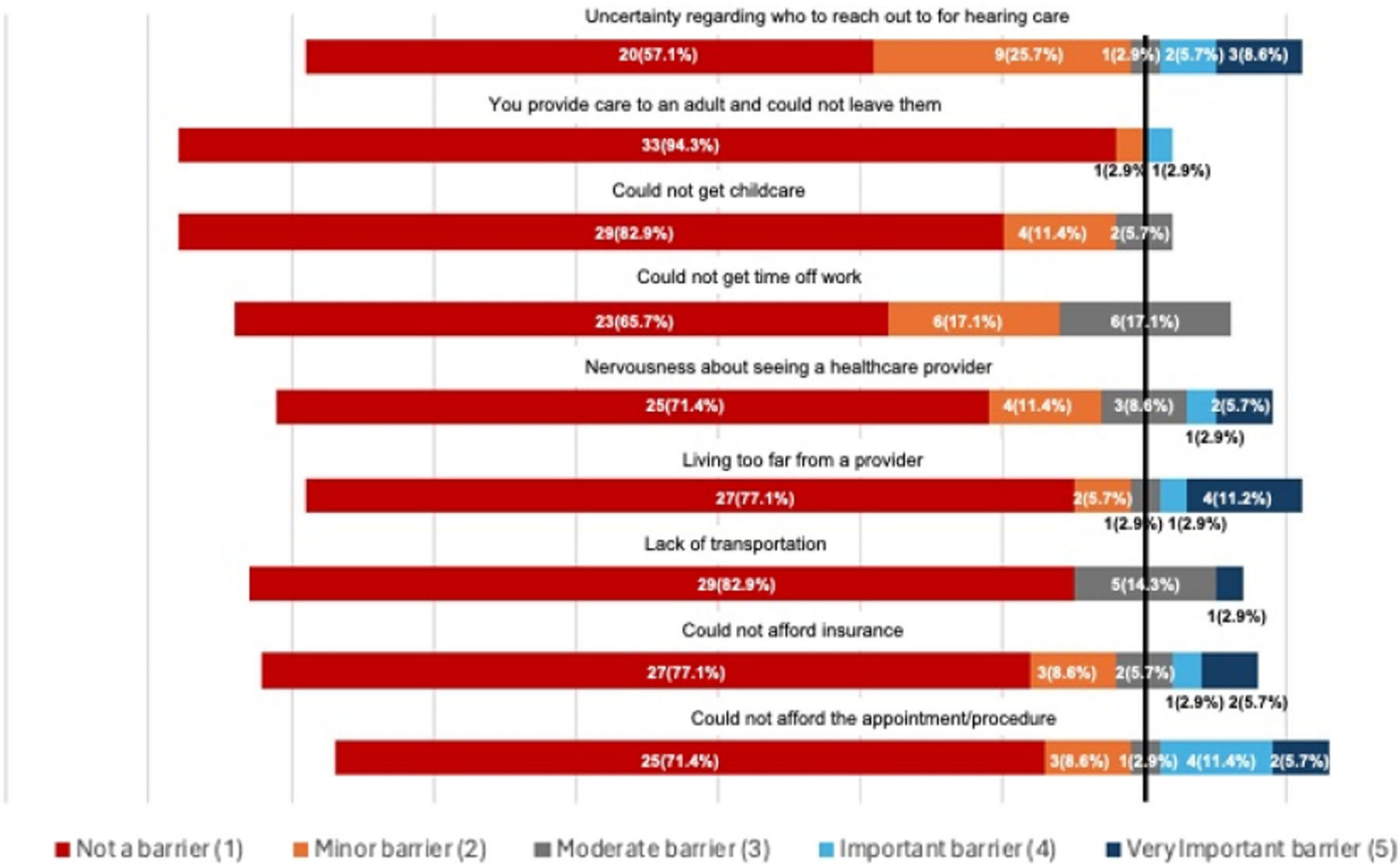
Bar graph of importance of barriers to care. *Importance is described by response to the query “Please rate how significant was each of the following barriers of care to getting hearing loss care.”

Figure 4 describes the relative importance of various barriers to care among Veterans who reported seeking any hearing care (for either hearing loss or tinnitus) and experiencing barriers to that care. Most reported barriers to care (minor barrier or greater), included uncertainty regarding who to reach out to for hearing care (42.9%), inability to get time off of work (34.3%), and nervousness about seeing a healthcare provider (28.6%).

**Figure 5:**
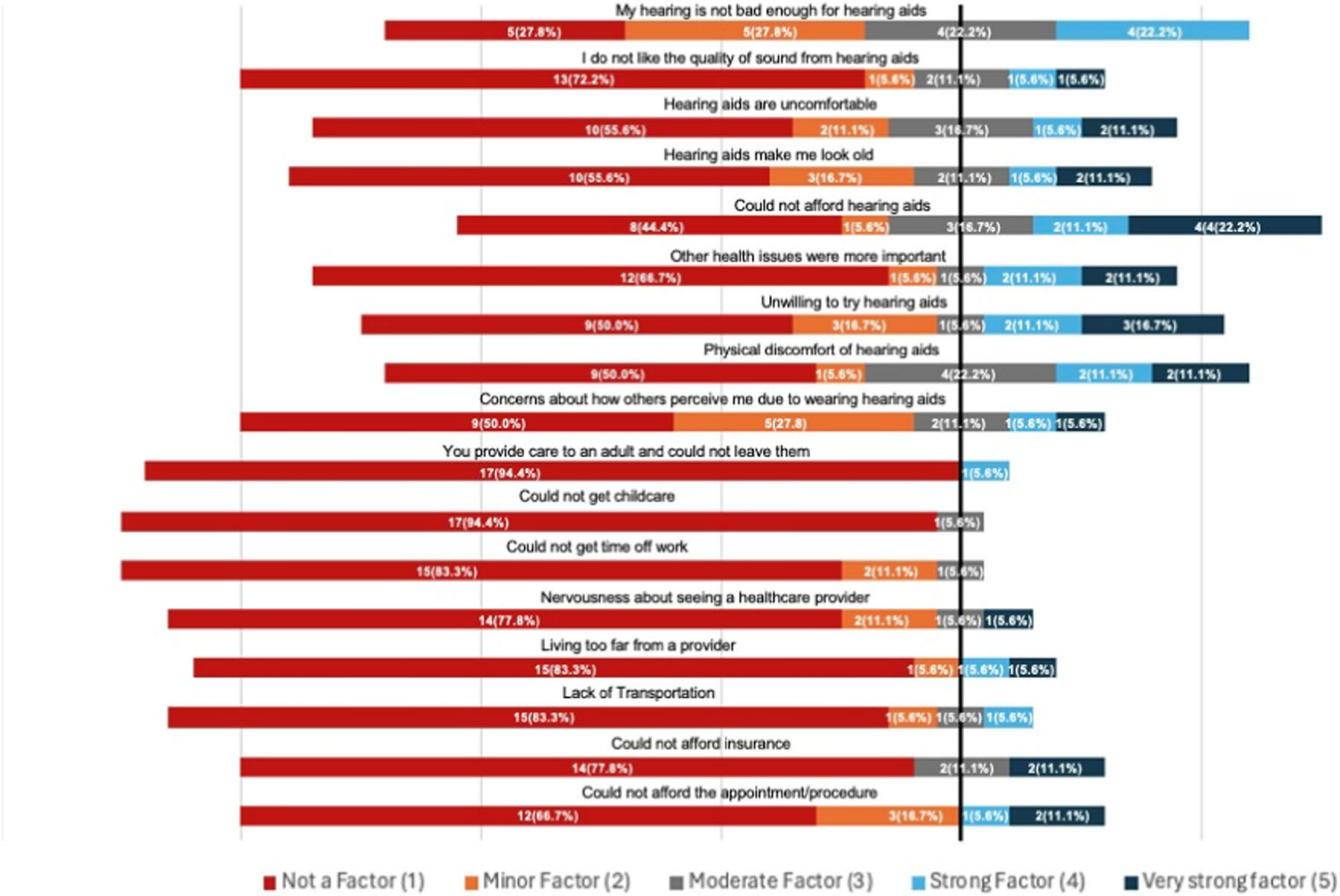
Reasons for declining recommended hearing aid*. *Reasons for declining hearing aids were described by response to the query “Please rate how important each factor was in your decision to not get hearing aids.”

Among 92 individuals who received a recommendation from a physician to get a hearing aid, 18 (19.6%) refused to get a hearing aid. Figure 5 describes most common reasons for refusal among this group. Reasons that most commonly played a factor in declining hearing aids (minor factor or greater) included reporting hearing was not bad enough for a hearing aid (72.2%), inability to afford hearing aids (55.6%), concerns about how others perceive me due to wearing hearing aids (50.0%), unwillingness to try hearing aids (50.0%), and physical discomfort of hearing aids (50.0%). Of note, inability to afford hearing aids had the highest percentage report of being a very strong factor (22.2%).

## Discussion

In this study, we explored the current use of hearing care among US veterans reporting hearing loss and/or tinnitus and their potential facilitators and barriers to hearing care. This study reconfirmed prior research demonstrating hearing loss and tinnitus are very common among US Veterans. Many have sought medical care but faced challenges in accessing hearing care. This study further explored motivating factors and barriers to hearing care among US veterans. Facilitators for hearing care were primarily internal, including significance of hearing and tinnitus problems, the importance of hearing in individuals' daily lives, and belief in an ability to fix those hearing problems. However, barriers were a mix of internal and external problems, including lack of awareness of how to seek hearing care, living too far from a provider, inability to afford appointments and procedures, and potentially lack of awareness of treatment options. Findings from this study on motivations, barriers, and adherence to hearing care offer initial insights for developing strategies to promote hearing health among US veterans.

While the high prevalence and morbidity of hearing loss and tinnitus among US veterans has been reported consistently in prior literature,^26^, ^27^, ^28^ exploration of hearing healthcare access among this population has been limited. Specifically, such studies have not adequately investigated all aspects of healthcare access as previously described,^13^ including motivations, barriers, and adherence to treatment recommendations. First, regarding motivation to access care, studies among other populations have identified self‐recognition of a hearing problem^29^ and perception of a benefit to receiving care^30^ to be primary motivating factors. Studies among veterans are lacking, but two qualitative studies identified severity in tinnitus and ability to improve their medical issues as significant motivators.^31^, ^32^

Overall, our findings demonstrated various external and internal barriers to hearing care among veterans. First, there are geographic disparities in ability to access hearing healthcare for veterans,^16^, ^33^ which is consistent with our finding that living far from a provider was one of the more important barriers explored. While some studies have identified knowledge and healthcare literacy as factors affecting ability to access hearing healthcare,^34^ our study suggests that this factor may be similarly important to the other discussed factors above. Inability to afford hearing aids was also a primary determinant of lack of adherence to hearing aid recommendations among US veterans—another external barrier to hearing care. Internal barriers also exist, including perceptions that hearing loss is not severe enough to warrant hearing aids despite professional recommendations, reluctance to try hearing aids, and concerns about how wearing hearing aids may be perceived by others. Prior studies have identified stigma as a significant barrier to seeking healthcare among veterans.^35^, ^36^ In the general population, increased hearing aid utilization has been associated with a greater perceived hearing deficit,^37^ a positive and open mindset towards hearing aids,^37^ as well as reduced cost.^38^ These findings highlight the importance of making hearing care more affordable and accessible, while also addressing the stigma associated with their use.

The experiences expressed by US Veterans in this study suggest potential changes that could improve hearing care for veterans. As previously demonstrated, while hearing care is generally provided for free by VA sites, there are reports of difficulty in obtaining hearing aids for many with significant variability in treatment for hearing loss and tinnitus among VA sites.^17^, ^18^, ^39^ Our finding that the average individual reported approximately a decade of hearing loss before receiving care is in line with previous reports of 7 to 10 years among a more general population, suggesting that current care access for veterans is insufficient.^40^, ^41^, ^42^ With uncertainty on how to reach out to for hearing care being one of the most reported barriers to care among veterans, one potential solution for improved access would be routine hearing screening for this at‐risk population. Unfortunately, the update from the US Preventive Service Task Force from 2021 found that the evidence is insufficient to recommend hearing screening among asymptomatic older adults 50 years or older.^4^ While a previous randomized controlled trial of hearing screening among US male veterans conducted in 2003 showed an equally low hearing aid use rate of less than 10% in both those who received hearing screening and those who did not,^43^ new studies are needed to assess the long‐term impact of hearing screening across different risk groups. These studies should aim to better understand the role of hearing screening in various age groups with extended follow‐up periods. To address the reported cost barriers and long travel distances to providers for hearing care treatment, it may be beneficial to allow veterans to access services in community settings in addition to the VA system. Finally, education on the importance of hearing loss, tinnitus, and treatment options may further improve hearing care access for veterans, as many reported knowledge of treatment options and limited confidence in ability to fix their hearing problem, particularly in the case of tinnitus.

This study has limitations. Although veteran status was reported by ResearchMatch, this—as well as hearing loss and/or tinnitus—were documented via self‐report and may not be accurate. Audiometric characteristics and physician records were not available for inclusion in this study. The utilization of multiple‐choice options limits the variety of perspectives we can learn about barriers to healthcare among veterans, though themes explored were informed by thorough literature review. Future studies may utilize qualitative methods to understand the broader themes for this topic. Additionally, a limitation of this study is its retrospective design, which could result in recall bias. While participants are drawn from an NIH‐supported pool of research participants, the sample size was overall small with low response rate. Our sample reported higher education levels compared to the broader veteran population. We acknowledge that our findings may not be representative of the average US veteran, who may face different barriers to accessing care. Additionally, there is a possibility that respondents with extreme views on hearing care may have been more likely to participate, introducing another source of bias. Due to these factors, the results are not generalizable to other settings. Future studies could improve generalizability through a survey at VA hospitals/clinics. Still, our study provides an initial step in exploring motivation, barriers, and adherence to hearing care among US veterans and brings new hypotheses about the relative contributions of various factors to each component of hearing care access for veterans. Future studies should further explore hypotheses generated from this study with the end goal of improving hearing care access for this at‐risk population.

## Conclusion

This study explored the current state of hearing care utilization among US veterans, along with the primary motivators, barriers, and factors affecting adherence regarding hearing care. It revealed unique insights into the relative importance of various factors affecting access to hearing care, such as a belief in ability to fix hearing problems, uncertainty on how to obtain hearing care, and the affordability of services. Further discussions with various stakeholders are needed to investigate how changes in policies and education can improve access to hearing care among US veterans.

## Author Contributions

**Tyler J. Gallagher**, conception and design of work, data acquisition and analysis, interpretation of data, drafting of manuscript, critical revision; **Kaitlin Hori**, Data acquisition and analysis, interpretation of data, drafting of manuscript, critical revision; **Janet S. Choi**, conception and design of work, data acquisition and analysis, interpretation of data, drafting of manuscript, critical revision.

## Disclosures

### Competing interests

None.

### Funding source

Academic departmental funding was utilized to support this study.

## Supporting information

Supporting information.
