# Supplementary material for: Access to Hearing Healthcare and Barriers Among United States Veterans: A Survey‐Based Study
Source: OTO Open. 2025 Jan 9;9(1):e70055. doi: 10.1002/oto2.70055 (PMC11714147; doi:10.1002/oto2.70055)
Supplement: Supplementary file 1 — Supporting information. [file OTO2-9-e70055-s001.pdf]

Hearing Access Survey

Please complete the anonymous survey below. Once you submit the survey, you will receive a link to enter your email for a $50 VISA Gift Card raffle.

Thank you!

What is your age in years?

(Place a mark on the scale above)

What is your gender?

- ◯ Male
- ◯ Female
- ◯ Other
- ◯ Prefer not to answer

What is your marital status?

- ◯ Married
- ◯ Single
- ◯ Long-term partnership
- ◯ Separated/Divorced
- ◯ Other/Prefer not to respond

What is your race/ethnicity?

- ◯ White/Caucasian, Non-Hispanic
- ◯ Hispanic/Latino
- ◯ Black/African American
- ◯ Asian American
- ◯ Other

What is your highest level of education?

- ◯ Some secondary/high school
- ◯ High school degree
- ◯ Some college
- ◯ College graduate
- ◯ Graduate school degree
- ◯ Doctorate degree
- ◯ Prefer not to respond

What is you annual household income?

- ◯ Less than $25,000
- ◯ $25,000-49,999
- ◯ $50,000-74,999
- ◯ $75,000-99,999
- ◯ $100,000-149,999
- ◯ $150,000-199,999
- ◯ $200,000 or greater
- ◯ Prefer not to respond

What is your primary healthcare insurance type?

- ◯ VA health plan or TRICARE
- ◯ Medicare
- ◯ Medicaid
- ◯ Private/Other employer-sponsored insurance
- ◯ No insurance

Hearing Handicap Inventory - This set of questions ask about your hearing.

The purpose of this scale is to identify problems your hearing loss may be causing you. Please select NO, SOMETIMES, or YES for each question. If you wear hearing instruments, please answer the way you hear without hearing instruments.

	No	Sometimes	Yes
Does a hearing problem cause you to feel embarrassed when you meet new people?	○	○	○
Does a hearing problem cause you to feel frustrated when talking to a member of your family?	○	○	○
Does a hearing problem cause you difficulty hearing/understanding coworkers, clients or customers?	○	○	○
Do you feel handicapped by a hearing problem?	○	○	○
Does a hearing problem cause you difficulty when visiting friends, relatives, or neighbors?	○	○	○
Does a hearing problem cause you difficulty at the movies or in theaters?	○	○	○
Does a hearing problem cause you to have arguments with family members?	○	○	○
Does a hearing problem cause you difficulty when listening to television or radio?	○	○	○
Do you feel that any difficulty with your hearing limits/hampers your personal or social life?	○	○	○
Does a hearing problem cause you difficulty when in a restaurant with relatives or friends?	○	○	○

Tinnitus handicap inventory - This section will ask questions about your experiences with tinnitus (ringing or buzzing in your ears).

The purpose of this scale is to identify problems tinnitus may be causing you.

	No, not a problem	Yes, a small problem	Yes, a moderate problem	Yes, a big problem	Yes, a very big problem
Over the last week, tinnitus kept me from sleeping.	◯	◯	◯	◯	◯
Over the last week, tinnitus kept me from concentrating on reading.	◯	◯	◯	◯	◯
Over the last week, tinnitus kept me from relaxing.	◯	◯	◯	◯	◯
Over the last week, I couldn't get my mind off of tinnitus.	◯	◯	◯	◯	◯

Have you ever had trouble with hearing loss?
◯ Yes
◯ No

Have you ever had trouble with tinnitus (ringing, roaring, or buzzing) in your ears?
◯ Yes
◯ No

Have you ever been diagnosed with hearing loss by a professional (e.g. any physician)?
◯ Yes
◯ No

Have you ever been diagnosed with tinnitus by a professional (e.g. any physician)?
◯ Yes
◯ No

Have you ever had a hearing test (audiogram)?
◯ Yes
◯ No

How long ago was your most recent audiogram (years)?

(Select 0 if less than 1 year ago)

(Place a mark on the scale above)

How long ago did you first notice hearing loss (years)?

(Select 0 if less than 1 year ago)

(Place a mark on the scale above)

How long ago did you first notice tinnitus (years)?

(Select 0 if less than 1 year ago)

(Place a mark on the scale above)

Have you ever sought medical care for hearing loss?
◯ Yes
◯ No

How long ago did you first seek medical care for hearing loss (years)?

(Select 0 if less than 1 year ago)

(Place a mark on the scale above)

Have you ever sought medical care for tinnitus?

○ Yes
○ No

How long ago did you first seek medical care for tinnitus (years)?

(Select 0 if less than 1 year ago)

(Place a mark on the scale above)

Hearing Care - This section asks questions about reasons you chose to seek care for your hearing loss and/or tinnitus.

Please rate how much each factor contributed to your decision to seek care for hearing loss and/or tinnitus (Ignore if no applicable questions are below)

	Not a factor	Mild factor	Moderate factor	Strong factor	Very strong factor
Degree of hearing loss	○	○	○	○	○
Degree of tinnitus	○	○	○	○	○
Difficulty hearing others	○	○	○	○	○
Psychological strain of hearing issue	○	○	○	○	○
Belief in ability to fix hearing problem	○	○	○	○	○
Importance of hearing in your life	○	○	○	○	○
Family/social support or encouragement	○	○	○	○	○
Appointment convenience	○	○	○	○	○

Please describe any other factors related to your motivation to get hearing care.

____

Did you experience any challenges or barriers to receiving care for hearing loss?

○ Yes
○ No

Did you experience any challenges or barriers to receiving care for tinnitus?

○ Yes
○ No

Barriers to hearing care - This section asks questions about your experience with barriers to hearing loss or tinnitus care.

Please rate how significant was each of the following barriers of care to getting hearing loss care (Ignore if no applicable questions are below)

	Not a barrier	Minor barrier	Moderate barrier	Important barrier	Very important barrier
Couldn't afford the appointment/procedure	◯	◯	◯	◯	◯
Couldn't afford insurance	◯	◯	◯	◯	◯
Lack of transportation	◯	◯	◯	◯	◯
Living too far from a provider	◯	◯	◯	◯	◯
Nervousness about seeing a healthcare provider	◯	◯	◯	◯	◯
Couldn't get time off work	◯	◯	◯	◯	◯
Couldn't get child care	◯	◯	◯	◯	◯
You provide care to an adult and could not leave them	◯	◯	◯	◯	◯
Uncertainty regarding who to reach out to for hearing care	◯	◯	◯	◯	◯

Please describe any other significant barriers to getting hearing care.

____

Has a physician ever recommended that you get a hearing assist device (e.g. hearing aid, cochlear implant, etc.)?

◯ Yes - hearing aid was recommended
◯ Yes - cochlear implant or other device was recommended
◯ No

Did you get a hearing aid?

◯ Yes
◯ No

Do you still use the hearing aid?

◯ Yes
◯ No

Hearing aid decision - Questions in this section will ask about reasons for your decision to decline a hearing aid.

Please rate how important each factor was in your decision to not get a hearing aid.

	Not a factor	Mild factor	Moderate factor	Strong factor	Very strong factor
Couldn't afford the appointment/procedure	◯	◯	◯	◯	◯

Couldn't afford insurance	○	○	○	○	○
Couldn't afford hearing aids	○	○	○	○	○
Lack of transportation	○	○	○	○	○
Living too far from a provider	○	○	○	○	○
Nervousness about seeing a healthcare provider	○	○	○	○	○
Couldn't get time off work	○	○	○	○	○
Couldn't get child care	○	○	○	○	○
You provide care to an adult and could not leave them	○	○	○	○	○
Concerns about how others perceive me due to wearing hearing aids	○	○	○	○	○
Physical discomfort of hearing aids	○	○	○	○	○
Unwilling to try hearing aids	○	○	○	○	○
Other health issues were more important	○	○	○	○	○
Hearing aids make me look old	○	○	○	○	○
Hearing aids are uncomfortable	○	○	○	○	○
I do not like the quality of sound from hearing aids	○	○	○	○	○
My hearing is not bad enough for hearing aids	○	○	○	○	○

Please describe any other important factors in your decision to not get a hearing aid. ________________________________

Did you get a cochlear implant or other device?
○ Yes
○ No

Cochlear implant decision - Questions in this section will ask about reasons for your decision to decline a cochlear implant.

Please rate how important each factor was in your decision to not get a cochlear implant.

	Not a factor	Mild factor	Moderate factor	Strong factor	Very strong factor
Couldn't afford the appointment/procedure	○	○	○	○	○
Couldn't afford insurance	○	○	○	○	○
Couldn't afford cochlear implant	○	○	○	○	○
Lack of transportation	○	○	○	○	○

Living too far from provider site	○	○	○	○	○
Nervousness about seeing a healthcare provider	○	○	○	○	○
Couldn't get time off work	○	○	○	○	○
Couldn't get child care	○	○	○	○	○
You provide care to an adult and could not leave them	○	○	○	○	○
Concerns about how others perceive me due to wearing a cochlear implant	○	○	○	○	○
Concern about surgery and risks	○	○	○	○	○
Concern for lack of improvement in hearing	○	○	○	○	○
Concern for lack of improvement in communication	○	○	○	○	○
Concern for loss of ability to appreciate music	○	○	○	○	○
Other health issues were more important	○	○	○	○	○

Please describe any other important factors in your decision to not get a cochlear implant or other device. ______________________________________

Hearing treatments - The questions below will ask about your familiarity with various treatments for hearing loss and/or tinnitus.

How familiar are you of the following treatments for hearing?

	Very unfamiliar	Somewhat unfamiliar	Neither familiar or unfamiliar	Somewhat familiar	Very familiar
Prescription hearing aids	○	○	○	○	○
Over the counter hearing aids	○	○	○	○	○
Cochlear implants	○	○	○	○	○
Bone anchored hearing aids	○	○	○	○	○
